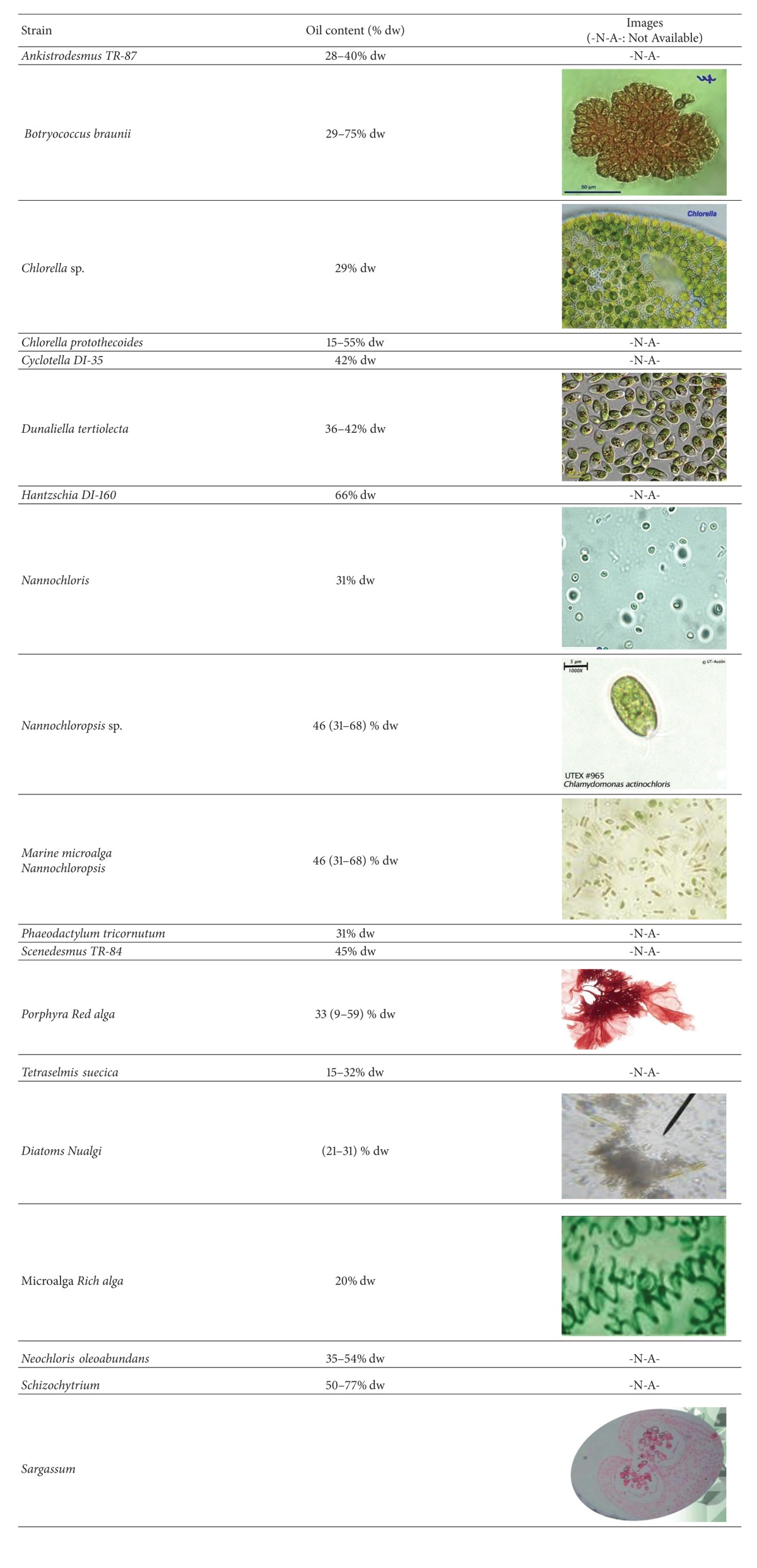# Algae Oil: A Sustainable Renewable Fuel of Future

**DOI:** 10.1155/2014/272814

**Published:** 2014-05-05

**Authors:** Monford Paul Abishek, Jay Patel, Anand Prem Rajan

**Affiliations:** School of Bio Sciences and Technology, VIT University, Vellore, Tamil Nadu 632014, India

## Abstract

A nonrenewable fuel like petroleum has been used from centuries and its usage has kept on increasing day by day. This also contributes to increased production of greenhouse gases contributing towards global issues like global warming. In order to meet environmental and economic sustainability, renewable, carbon neutral transport fuels are necessary. To meet these demands microalgae are the key source for production of biodiesel. These microalgae do produce oil from sunlight like plants but in a much more efficient manner. Biodiesel provides more environmental benefits, and being a renewable resource it has gained lot of attraction. However, the main obstacle to commercialization of biodiesel is its cost and feasibility. Biodiesel is usually used by blending with petro diesel, but it can also be used in pure form. Biodiesel is a sustainable fuel, as it is available throughout the year and can run any engine. It will satisfy the needs of the future generation to come. It will meet the demands of the future generation to come.

## 1. Introduction

Oil depletion is the degradation in oil production of a well or oil field. A 2010 study published in the journal, Energy Policy by researchers from Oxford University, predicted that demand would surpass supply by 2015, unless forced by strong recession pressures caused by reduced supply or government interference [1]. It relates to long-term degradation in the availability of petroleum. On an average, human utilizes fossil fuels which results in the release of 29 gigatonnes CO each year. These figures point towards Hubbert's peak theory according to which peak oil is the point in time when the maximum rate of petroleum extraction is reached, after which the rate of production is expected to enter terminal decline [2]. This critical situation has led to the emergence of an eco-friendly, alternative fuel biodiesel. According to United States Environmental Protection Agency, the volume requirement of the biomass based diesel in 2013 is 1.28 million gallons which accounts for 1.13% of the total renewable fuels. This, combined with growing demand, significantly increases the worldwide prices of petroleum derived products. Most important concerns are the availability and price of liquid fuel for transportation [3].

In recent years, the use of biofuels has shown manifold global growth in the transport sector due to the policies concentration on achieving energy conservation and the avoidance of excess or extremes of GHG (greenhouse gases) emissions [4]. The 1st generation biofuels which are extracted from oil crops like rapeseed oil, sugarcane, sugar beet, and maize [5] including vegetable oils and animal fat using conventional technology have attained profitable levels of production [6]. But the use of 1st generation biofuels has raised questions and controversies due to their impact on the global food market and food security [7]. For example, the demand for biofuels may impose additional pressure on natural resource base, with potentially harmful surrounding and social concerns [8].

Energy shortage refers to the crisis of energy resources to an economy. There has been a massive uplift in the global demand for energy in recent years as a result of industrial development and population growth. Since the early 2000s, the demand for energy, especially from liquid fuels, and limits on the rate of fuel production have created such a stage leading to the current energy crisis. The cause may be overconsumption, aged infrastructure, choke point disruption or crisis at oil refineries, and port facilities that confine fuel supply.

In this paper, we have focused on addressing the global oil shortage by replacing nonrenewable source of oil reservoir by evergreen renewable natural source, algae oil.

Microalgae cover unicellular and simple multicellular microorganisms, including prokaryotic microalgae that are cyanobacteria (*chloroxybacteria*) and eukaryotic microalgae for example, green algae (*chlorophyta*), and diatoms (*bacillariophuta*). These microalgae are beneficial as they are capable of all year production [9]; they grow in aqueous media and hence need less water than terrestrial crops [10]; microalgae can be cultivated in brackish water on noncultivated land [11] and they have rapid growth potential and have oil content up to 20–50% dry weight of biomass [12, 13]. Unlike other biodeisel corps microalgae does not require herbicides or pesticides [13], microalgae also produce beneficial coproducts such as proteins and residual biomass after oil extraction, which can be used as feed or fertilizer or can be fermented to produce ethanol or methane [14]; the oil yield, can be significantly increased by varying growth conditions to modulate biochemical composition of algal biomass [15]. They also produce beneficial coproducts such as proteins and residual biomass after oil extraction, which can be used as feed or fertilizer or can be fermented to produce ethanol or methane [16]; the oil yield can be significantly increased by varying growth conditions to modulate biochemical composition of algal biomass [17].

The algal biofuel technology includes selection of specific species for production and extraction of valuable co-products [18]. The algaes are bioengineered for achieving advanced photosynthetic efficiencies through continued development of production system [19]. Challenges include, only single species cultivation techniques which are developed so far and are recommended to follow globally, but mixed culture may yield more algae oil than mono culture [20]. Algae oil may be less economically which includes techniques such as water pumping, CO_2_ transmission, harvesting and extraction [21]. Fatal compounds such as NO_*x*_ and SO_*x*_ are produced in high concentrations as fuel gases, which are not environmental friendly [22].

Microalgae are sunlight-driven cell factories that transform carbon dioxide to potential biofuels, foods, feeds, and high-value bioactive. In addition, these photosynthetic microorganisms are useful in bioremediation applications and as nitrogen fixing biofertilizers. This review focuses on microalgae as a potential basis of biodiesel.

The idea of using microalgae as a source of fuel is not novel, but it is now taken seriously because of the increasing price of petroleum and, more significantly, the emerging issues about global warming and greenhouse effect that is associated with incinerating fossil fuels. Thus, several companies are involved in the production of algal fuel in order to decrease global warming and greenhouse effect. Biodiesel is an established fuel. In the United States, biodiesel is produced mainly from soybeans [23]. Other origins of commercial biodiesel include canola oil, animal fat, palm oil, corn oil [24], and waste cooking oil. Microalgae offer several different kinds of renewable biofuels [25].

The yields of different oil producing feedstock can be explained, as shown in Table 1.

### 1.1. Unavailability of Resources

The feedstock is not available for the biodiesel production as it is unethical to use these cash crops for fuel while the world is witnessing food shortage. The primary cause for global food shortage may be due to overconsumption, overpopulation, and overexploitation.

### 1.2. Peak Oil

Peak oil is the point where maximum extraction of petroleum is reached, after which the rate of production enters decline stage [28]. The invention of new fields, the development of new production techniques, and the misuse of eccentric supplies have resulted in productivity levels, which endure to increase. Peak oil is often confused with oil depletion; peak oil is the point of maximum extraction, while depletion indicates the period of falling in production and supply.

## 2. Sources of Biodiesel

A variety of oils can be used to produce biodiesel. These include the following.

### 2.1. Virgin Oil Feedstock

Rapeseed and soybean oils are most commonly used, mostly in U.S [29]. They also can be obtained from* Pongamia*, field pennycress, Jatropha, and other crops such as mustard, jojoba, flax, sunflower, palm oil, coconut, and hemp. Several companies in various sectors are piloting research on* Jatropha curcas*, a poisonous shrub-like tree that produces seeds, considered by many to be a feasible source of biodiesel feedstock oil [30].

### 2.2. Waste Vegetable Oil (WVO)

Vegetable oil is an alternative fuel source for diesel engines and for heating oil burners. The viscosity of the vegetable oil plays an important role in the atomization of fuel for engines designed to burn diesel fuel; otherwise, it causes improper combustion and causes engine collapse. The most important vegetable oils used as fuel are rapeseed oil (also known as canola oil, which is mostly used in the United States and Canada). In some places of the United States, the use of sunflower oil as fuel tends to increase [31]. Some island nations use coconut oil as fuel to lower their expenses and their dependence on imported fuels. The annual vegetable oil recycled in the United States, as of 2000, was in excess of 11 billion liters (2.9 billion U.S. gallons), mainly produced from industrial deep fryers in potato processing plants, snack factories and fast food restaurants. If all those 11 billion liters could be recycled, it could replace the energy equivalent amount of petroleum [32]. Other vegetable oils which can be used as fuel are cottonseed oil, peanut oil, and soybean oil [31].

### 2.3. Animal Fats

Animal fats are the by-product of meat production and cooking. These include tallow, lard, yellow grease, chicken fat, and the by-products of the production of omega-3 fatty acids from fish oil [33]. Oil yielding Plants like* Salicornia bigelovii*, a halophyte, is harvested using brackish water in coastal areas where conventional crops are not feasible to be grown. The oil from* Salicornia bigelovii* equal to the yields of soybeans and other oilseeds grown by freshwater irrigation [34].

Multifeedstock biodiesel facilities produce high standard animal-fat based biodiesel. Currently, a 5-million-dollar plant is being built in the USA, with the objective of producing 11.4 million litres (3 million gallons) biodiesel from the evaluated 1 billion kg (2.2 billion pounds) of chicken fat produced annually at the local Tyson poultry plant [33].

### 2.4. Sewage Sludge

Sludge refers to the unused, semisolid material left from industrial wastewater or sewage treatment processes. It can also refer to the settled suspension obtained from drinking water treatment and other industrial processes. Sludge is generally produced by a poorly designed or defective ventilation system, low engine operating temperatures or the presence of water in the oil. The sewage-to-biofuel field process is developing interest from major companies like Waste Management and startups like InfoSpi, which are challenging that renewable sewage biodiesel can become modest with petroleum diesel on price [35].

## 3. Algal Fuel

Algae fuel or algal biofuel is another form of fossil fuel that uses microalgae as its source of natural deposits [36]. Some of the unique characteristics of algal fuels are as follows: they can be grown with negligible impact on fresh water resources [37], they can be synthesized using ocean and wastewater, and they are biodegradable and relatively harmless to the environment if spilled [38, 39]. Algae cost more per unit mass due to the high capital and production costs.

The US Department of Energy's Aquatic Species Program, 1978–1996, was engrossed in biodiesel from microalgae. The final report recommended that biodiesel could be the only feasible method to produce enough fuel to change current world diesel consumption [40]. Algal fuel is highly favorable and feasible related to other biofuels, as they do not have to produce structural compounds and they can convert higher fractions of biomass to oil compared to other cultivated crops [41].

Studies display that some species of algae have the ability to produce up to 60% of their dry weight in the form of oil. Because the cells grow in aqueous suspension, where they have more effective access to water, CO_2_ and nutrients are capable of producing large amounts of biomass and usable oil in either high rate algal ponds or photobioreactors (Table 2).

**Figure 1:**
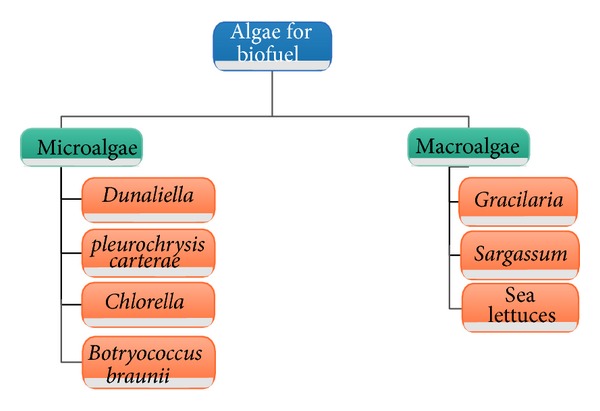
Classified Algae used for biodiesel production.

Regional cultivation of microalgae and producing biofuels will ensure economic benefits to rural communities [42].  Figure 1 differentiates algae based on the species and their size range (few micrometers (*μ*m) to a few hundreds of micrometers), as macroalgae and microalgae are used in the production of biodiesel.

## 4. Advantages of Algal Fuel over Other Sources

### 4.1. Easy Growth Rate

**Figure 2:**
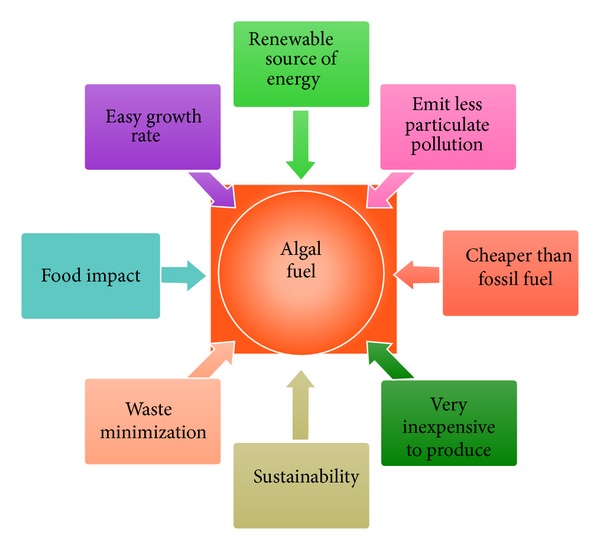
Advantages of algal fuel.

One of the most important advantages of using algae as the source is that it can be grown very easily. Wastewater which normally hinders plant growth is very effective in growing algae. The growth rate of algae is 20–30 times faster than other conventional crops like* Jatropha* [43]. A diagram of the advantages of algal fuel is presented in Figure 2.

### 4.2. Food Impact

Many outmoded feedstocks for biodiesel, such as corn and palm, can also be used as feed for livestock on farms, as well as reliable source of food for humans. Because of this, using them as biofuel decreases the amount of food available for both, and this causes an increased expense for both the food and the fuel produced. By using algae as a source of biodiesel can make this issue in a number of ways. First, algae are not used as a primary food source for humans, meaning that it can be used distinctly for fuel and there would be less impact on the food industry [44]. Second, many of the waste-product sources produced during the processing of algae for biofuel can be used as an efficient animal feed. This is an efficient way to minimize waste and a much cheaper remedy to the more traditional corn or grain based feeds [45].

### 4.3. Waste Minimization

Growing algae have been shown to have various environmental benefits, proved to be the environmental friendly biofuel [43, 45]. Because of this, it ensures that contaminated water does not mix with the lakes and rivers that presently supply our drinking water. In addition to this, the ammonia, nitrates, and phosphates that would generally render the water unsafe actually serve as excellent nutrients for the algae [48].

## 5. Production

### 5.1. Algae Cultivation

Algae are typically found growing in ponds, waterways, or other wetlands which receive sunlight and CO_2_. Growth varies on many factors and can be enhanced for temperature, sunlight utilization, pH control, fluid mechanics, and more [49, 50]. Man-made production of algae tends to replicate the natural environments to achieve ideal growth conditions. Algae production systems can be organized into two distinct categories: open ponds and closed photo bioreactors. Open ponds are simple expanses of water sunken into the ground with some mechanism to deliver CO_2_ and nutrients with paddle wheels to mix with the algal broth. Closed photo bioreactors are a broad category referring to systems that are bounded and which allow more precise control over growth conditions and resource management.

### 5.2. Algae Biofilm

Biofilm formed by algae can be harvested easily using unit operations like filtering, scraping, size reduction, and drying. Photoreactors are used to produce high quality algae in either sessile from or mainly biofilm (attached form). Attached algae have produced more oil than planktonic form. The reason for high lipid content is due to alteration in the lipid metabolic pathway of attached algae resulting in change in the membrane fluidity of algae to make them attached to a substratum. For small-scale as well as large-scale production, the photoreactors are used wherein natural or synthetic light can be used to grow algae.

### 5.3. Algae Harvesting and Oil Extraction

Production of oil from algae is a straightforward process that consisted of growing the algae by providing necessary inputs for photosynthesis, harvesting, dewatering, and oil extraction. Energy in the form of photons is absorbed by the algae cells, which convert the inorganic compounds of CO_2_ and water into sugars and oxygen. The sugars are eventually converted into complex carbohydrates, starches, proteins, and lipids within the algae cells. In order to extract the valuable lipids, a series of steps must be undertaken to isolate the algae cells and oil.

**Figure 3:**
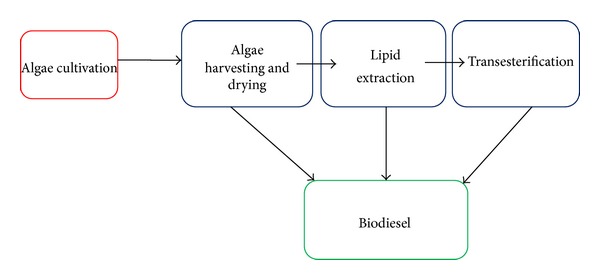
Algae growth and harvesting process [46].

**Figure 4:**
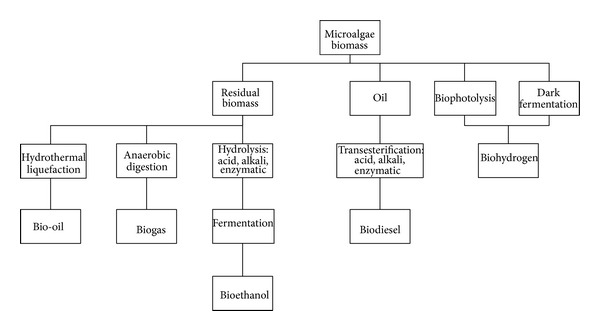
Principal Microalga biomass transformation processes for biofuel production [47].

A diagram of the overall growth and harvesting process is presented in Figure 3. The traditional process begins by separating the algae biomass from the water broth in the dewatering* stage using centrifuges, filtration, or flocculation techniques. Centrifuges collect biomass by spinning the algae-water broth so that water is flung away from the algae cells. Flocculation involves precipitating algae cells out of solution so that they can be concentrated and removed easily. Once the algae cells have been collected the oil must be removed from the cells. The oil can then be processed into biodiesel, jet fuel, ethanol, synthetic fuels, or other chemicals.  Figure 4 explains the overall microalga biomass transformation processes for biofuel production.

*Liquefaction (Dewatering). High content of water often exists in microalgae after harvesting which requires a great deal of energy to remove moisture in the algal cells in the period of pretreatment. Liquefaction has been developed to produce biofuel directly without the need of drying microalgae. Moreover, wet microalgae can provide hydrogen for hydrogenolysis [51].

### 5.4. Transesterification

Biodiesel is commonly produced by the transesterification of the vegetable oil, animal fat, or algal feedstock. There are several methods for carrying out this transesterification reaction including the collective batch process, supercritical processes, ultrasonic methods, and even microwave methods.

Chemically, transesterified biodiesel comprises a mix of mono-alkyl esters of long chain fatty acids. The most conjoint form uses methanol (converted to sodium methoxide) to produce methyl esters (commonly referred to as fatty acid methyl ester (FAME)) as it is the cheapest alcohol available; though ethanol can be used to form an ethyl ester (commonly referred to as fatty acid ethyl ester (FAEE)), biodiesel and higher alcohols such as isopropanol and butanol have also been used. Using alcohols of higher molecular weights improves the cold flow properties of the resulting ester, at the cost of a less efficient transesterification reaction. A lipid transesterification production process converts the base oil to the desired esters. Any free fatty acids (FFAs) in the base oil are either converted to soap or removed from the process, or they are esterified (yielding more biodiesel) using an acidic catalyst. After this processing, biodiesel has combustion properties very similar to those of petroleum diesel and can replace it in most present uses.

The methanol used in most biodiesel production processes is made by fossil fuel inputs. However, there are sources of renewable methanol synthesized using carbon dioxide or biomass as feedstock, making their production processes free of fossil fuels [52].

## 6. Conclusion

As justified here, microalgal biodiesel is technically feasible. It is the only renewable biodiesel that can potentially and methodically displace liquid fuels obtained from petroleum. Economics of producing microalgal biodiesel need to improvise substantially to make it competitive with petro diesel, but the level of improvement necessary appears to be possible. Producing low-cost microalgal biodiesel requires primarily improvements to algal biology through genetic and metabolic engineering. Use of the biorefinery concept and advances in photobioreactor engineering will further reduce the cost of production. In view of their much greater productivity than raceways, tubular photobioreactors are likely to be used in producing most of the microalgal biomass required for making biodiesel. Algae biofilm grown in photobioreactors provide a controlled environment that can be tailored to the specific demands of highly productive microalgae to attain a consistently good annual yield of oil.

## Figures and Tables

**Table 1:** Amount of oil produced by various feedstocks [26].

Feedstock	Liters/hectare
Castor	1413
Sunflower	952
Palm	5950
Soya bean	446
Coconut	2689
**Algae**	**100000**

**Table 2:** Algae species for alga oil and their typical oil content [27].